# Supplementary material for: Global hypo-methylation in a proportion of glioblastoma enriched for an astrocytic signature is associated with increased invasion and altered immune landscape
Source: eLife. 2022 Nov 22;11:e77335. doi: 10.7554/eLife.77335 (PMC9681209; doi:10.7554/eLife.77335)
Supplement: Figure 2—source data 1. [file elife-77335-fig2-data1.zip › Figure_2_source_data_1/Figure_2B/homerResults/motif32.similar.html]

motif32

## Information for motif32

A
G
T
C
A
C
T
G
A
C
G
T
A
C
G
T
A
G
T
C
A
C
T
G
A
C
G
T
A
C
T
G
  
Reverse Opposite:  

A
G
T
C
C
G
T
A
A
G
T
C
A
C
T
G
C
G
T
A
C
G
T
A
A
G
T
C
A
C
T
G
  

|  |  |
| --- | --- |
| p-value: | 1e-43 |
| log p-value: | -9.908e+01 |
| Information Content per bp: | 1.530 |
| Number of Target Sequences with motif | 67.0 |
| Percentage of Target Sequences with motif | 2.90% |
| Number of Background Sequences with motif | 1.6 |
| Percentage of Background Sequences with motif | 0.45% |
| Average Position of motif in Targets | 92.9 +/- 55.6bp |
| Average Position of motif in Background | 143.8 +/- 21.3bp |
| Strand Bias (log2 ratio + to - strand density) | 0.8 |
| Multiplicity (# of sites on avg that occur together) | 1.08 |
| Motif File: | file (matrix) reverse opposite |

### Similar de novo motifs found

|  |  |  |  |  |  |  |  |
| --- | --- | --- | --- | --- | --- | --- | --- |
| Rank | Match Score | Redundant Motif | P-value | log P-value | % of Targets | % of Background | Motif file |
| 1 | 0.680 | A C T G A C G T C G A T A G T C A C T G A T G C | 1e-28 | -65.681721 | 4.37% | 1.16% | motif file (matrix) |
| 2 | 0.714 | C G T A A G T C A C T G G T A C C G T A A G T C | 1e-25 | -59.172434 | 5.76% | 2.05% | motif file (matrix) |
| 3 | 0.663 | A T G C C A T G C T G A C A G T A T G C A C T G | 1e-23 | -53.249724 | 1.99% | 0.37% | motif file (matrix) |
| 4 | 0.710 | A C G T A G T C C G T A A G T C A C T G C G T A | 1e-13 | -31.126205 | 3.63% | 1.53% | motif file (matrix) |
| 5 | 0.722 | C G T A A G T C A C T G A C G T A C G T A G T C | 1e-9 | -22.656414 | 1.82% | 0.83% | motif file (matrix) |
| 6 | 0.674 | A G T C A G T C A C G T A C G T A G T C A C T G | 1e-9 | -21.972028 | 3.20% | 1.61% | motif file (matrix) |
